# Design of Epitope-Based Peptide Vaccine against Pseudomonas aeruginosa Fructose Bisphosphate Aldolase Protein Using Immunoinformatics

**DOI:** 10.1155/2020/9475058

**Published:** 2020-11-05

**Authors:** Mustafa Elhag, Ruaa Mohamed Alaagib, Nagla Mohamed Ahmed, Mustafa Abubaker, Esraa Musa Haroun, Sahar Obi Abd Albagi, Mohammed A. Hassan

**Affiliations:** ^1^Faculty of Medicine, University of Seychelles-American Institute of Medicine, Seychelles; ^2^Department of Pharmacies, National Medical Supplies Fund, Sudan; ^3^Faculty of Medical Laboratories Sciences, Al-Neelain University, Sudan; ^4^Faculty of Medical Laboratory Sciences, Sudan University of Science and Technology, Sudan; ^5^Faculty of Medical Pharmacology, Ahfad University for Women, Sudan; ^6^Department of Bioinformatics, DETAGEN Genetics Diagnostic Center, Kayseri, Turkey

## Abstract

*Pseudomonas aeruginosa* is a common pathogen that is responsible for serious hospital-acquired infections, ventilator-associated pneumonia, and various sepsis syndromes. Also, it is a multidrug-resistant pathogen recognized for its ubiquity and its intrinsically advanced antibiotic-resistant mechanisms. It usually affects immunocompromised individuals but can also infect immunocompetent individuals. There is no vaccine against it available till now. This study predicts an effective epitope-based vaccine against fructose bisphosphate aldolase (FBA) of *Pseudomonas aeruginosa* using immunoinformatics tools. The protein sequences were obtained from NCBI, and prediction tests were undertaken to analyze possible epitopes for B and T cells. Three B cell epitopes passed the antigenicity, accessibility, and hydrophilicity tests. Six MHC I epitopes were found to be promising, while four MHC II epitopes were found promising from the result set. Nineteen epitopes were shared between MHC I and II results. For the population coverage, the epitopes covered 95.62% worldwide excluding certain MHC II alleles. We recommend *in vivo* and *in vitro* studies to prove its effectiveness.

## 1. Introduction

*Pseudomonas aeruginosa* is a motile, nonfermenting, gram-negative opportunistic bacterium that is implicated in respiratory infections, urinary tract infections, gastrointestinal infections, keratitis, otitis media, and bacteremia in patients with compromised host defences (e.g., cancer, burn, HIV, and cystic fibrosis) [1]. Intensive care unit (ICU) hospitalized patients constitute one of the risk groups that are more susceptible to acquire pseudomonas infections as they may develop ventilator-associated pneumonia (VAP) and sepsis [2–4]. This organism is a ubiquitous and metabolically versatile microbe that flourishes in many environments and possesses many virulence factors that contribute to its pathogenesis [1]. According to data from Centers for Disease Control, *P. aeruginosa* is responsible for millions of infections each year in the community, 10–15% of all healthcare-associated infections, with more than 300,000 cases annually in the EU, USA, and Japan [5]. It is a common nosocomial pathogen [6, 7] that causes infections with a high mortality rate [8, 9] which is attributable to the organism that possesses an intrinsic resistance to many antimicrobial agents [10] and the development of increased, multidrug resistance in healthcare settings [11–13], both of which complicate antipseudomonal chemotherapy. As a result, it remains difficult to combat *P. aeruginosa* infections despite supportive treatments. Vaccines could be an alternative strategy to control *P. aeruginosa* infections and even reduce antibiotic resistance; however, no *P. aeruginosa* vaccine is currently available [14]. Döring and Pier represented that the serious obstacle to the development of a globally effective anti-P. aeruginosa vaccine is due to the antigenic variability of a microorganism that enables it to easily adapt to different growth conditions and escapes host immune recognition and to the high variability of the proteins among different *P. aeruginosa* strains and within the same strain, grown in diverse environmental conditions [15].

Contemporary, integrated genomics and proteomics approaches have been used to predict vaccine candidates against *P. aeruginosa* [16]. Although several vaccine formulations have been clinically tested, none has been licensed yet [15, 17]. The search for new targets or vaccine candidates is of high paramount. Bioinformatics-based approach is a novel platform to identify drug targets and vaccine candidates in human pathogens [18, 19]. Thus, the present study is aimed at designing an effective peptide vaccine against *P. aeruginosa* using computational approach through prediction of highly conserved T and B cell epitopes from the highly immunogenic protein fructose bisphosphate aldolase (FBA). This is the first study that predicts epitope-based vaccine from this moonlighting protein of *P. aeruginosa*. This technique has been successfully used by many authors to identify target vaccine candidates. These types of vaccines are easy to produce, specific, capable of keeping away any undesirable immune responses, reasonable, and safe when compared to the conventional vaccines such as killed and attenuated vaccines [20].

## 2. Materials and Methods

### 2.1. Protein Sequence Retrieval

A total of 20,201 strains of *Pseudomonas aeruginosa* FBA were retrieved in FASTA format from the National Center for Biotechnology Information (NCBI) database (https://ncbi.nlm.nih.gov) on May 2019. The protein sequence had a length of 354 with the name fructose-1,6-bisphosphate aldolase.

### 2.2. Determination of Conserved Regions

The retrieved sequences of *Pseudomonas aeruginosa* FBA were subjected to multiple sequence alignment (MSA) using the ClustalW tool of BioEdit Sequence Alignment Editor Software version 7.2.5 to determine the conserved regions. Also, molecular weight and amino acid composition of the protein were obtained [21, 22].

### 2.3. Sequenced-Based Method

The reference sequence (NP_249246.1) of *Pseudomonas aeruginosa* FBA was submitted to different prediction tools at the Immune Epitope Database (IEDB) Analysis Resource (http://www.iedb.org/) to predict various B and T cell epitopes. Conserved epitopes would be considered candidate epitopes for B and T cell [23].

### 2.4. B Cell Epitope Prediction

B cell epitope is the portion of the vaccine that interacts with B lymphocytes which are a type of white blood cell of the lymphocyte subtype. Candidate epitopes were analyzed using several B cell prediction methods from the IEDB (http://tools.iedb.org/bcell/) to identify the surface accessibility, antigenicity, and hydrophilicity with the aid of random forest algorithm, a form of unsupervised learning. The BepiPred linear prediction 2 was used to predict linear B cell epitope with the default threshold value 0.533 (http://tools.iedb.org/bcell/result/). The Emini surface accessibility prediction tool was used to detect the surface accessibility with the default threshold value 1.00 (http://tools.iedb.org/bcell/result/). The Kolaskar and Tongaonkar antigenicity method was used to identify the antigenicity sites of a candidate epitope with the default threshold value 1.032 (http://tools.iedb.org/bcell/result/). The Parker hydrophilicity prediction tool was used to identify the hydrophilic, accessible, or mobile regions with the default threshold value 1.695 [24–28].

### 2.5. T Cell Epitope Prediction MHC Class I Binding

T cell epitope is the portion of the vaccine that interacts with T lymphocytes. Analysis of peptide binding to the MHC (major histocompatibility complex) class I molecule was assessed by the IEDB MHC I prediction tool (http://tools.iedb.org/mhci/) to predict cytotoxic T cell epitopes (also known as CD8+ cell). The presentation of peptide complex to T lymphocyte undergoes several steps. The Artificial Neural Network (ANN) 4.0 prediction method was used to predict the binding affinity. Before the prediction, all human allele lengths were selected and set to 9 amino acids. The half-maximal inhibitory concentration (IC50) value required for all conserved epitopes to bind was a score less than 500 [29–35].

### 2.6. T Cell Epitope Prediction MHC Class II Binding

Prediction of T cell epitopes interacting with MHC class II was assessed by the IEDB MHC II prediction tool (http://tools.iedb.org/mhcii/) for helper T cell, which is known as CD4+ cell also. Human allele reference set was used to determine the interaction potentials of T cell epitopes and MHC class II allele (HLA DR, DP, and DQ). The NN-align method was used to predict the binding affinity. IC50 score values less than 100 were selected [36–39].

### 2.7. Population Coverage

The population coverage tool was selected to analyze the epitopes in the IEDB. This tool calculates the fraction of individuals predicted to respond to a given set of epitopes with known MHC restriction (http://tools.iedb.org/population/iedbinput). The appropriate checkbox for calculation was checked based on MHC I, MHC II separately, and a combination of both [40].

### 2.8. Homology Modelling

The 3D structure was obtained using RaptorX (http://raptorx.uchicago.edu), i.e., a protein structure prediction server developed by Peng and Xu's group, excelling at 3D structure prediction for protein sequences without close homologs in the Protein Data Bank (PDB). USCF Chimera (version 1.8) was the program used for visualization and analysis of molecular structure of the promising epitopes (http://www.cgl.uscf.edu/chimera) [41, 42].

## 3. Results

### 3.1. Amino Acid Composition

**Figure 1:**
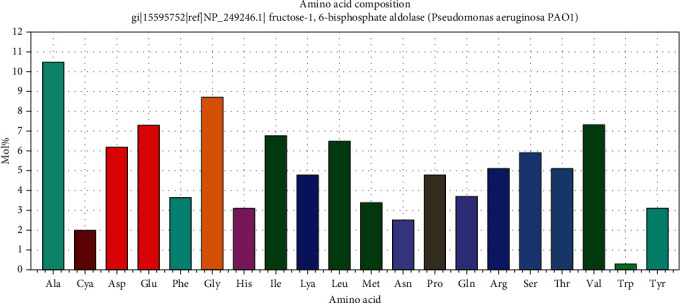
Amino acid composition for *Schistosoma mansoni* FBA using BioEdit software.

The amino acid composition for the reference sequence of *Pseudomonas aeruginosa* FBA is illustrated in Figure 1. Alanine and glycine were the most frequent amino acids (Table 1).

### 3.2. B Cell Epitope Prediction

**Figure 2:**
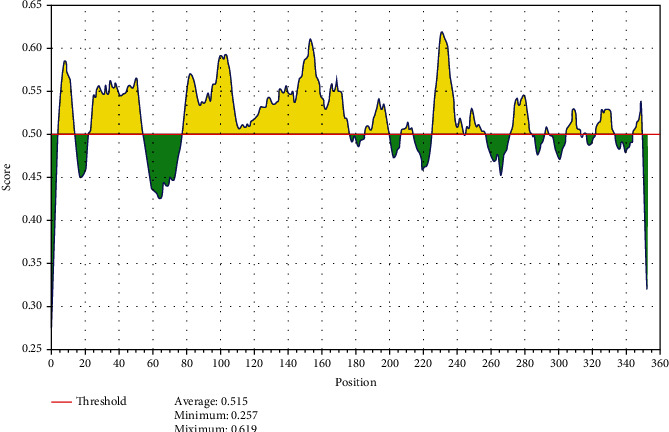
BepiPred linear epitope prediction; yellow areas above the threshold (red line) are proposed to be a part of B cell epitopes, and the green areas are not.

**Figure 3:**
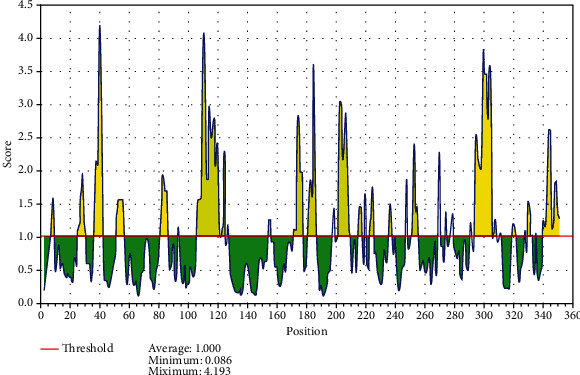
Emini surface accessibility prediction; yellow areas above the threshold (red line) are proposed to be a part of B cell epitopes, and the green areas are not.

**Figure 4:**
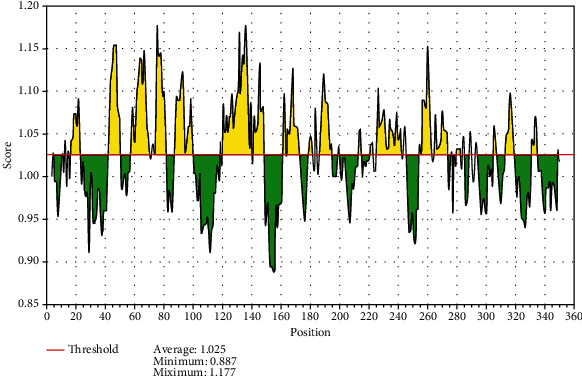
Kolaskar and Tongaonkar antigenicity prediction; yellow areas above the threshold (red line) are proposed to be a part of B cell epitopes, and green areas are not.

**Figure 5:**
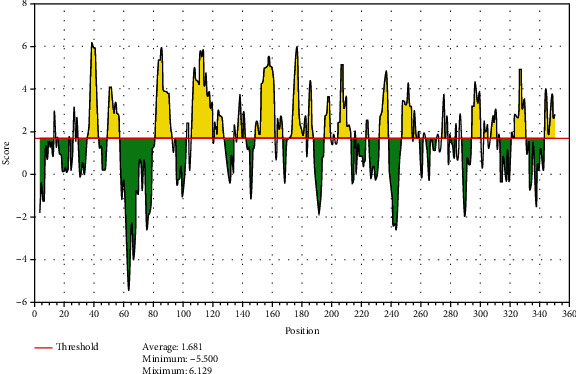
Parker hydrophilicity prediction; yellow areas above the threshold (red line) are proposed to be a part of B cell epitopes, and green areas are not.

**Figure 6:**
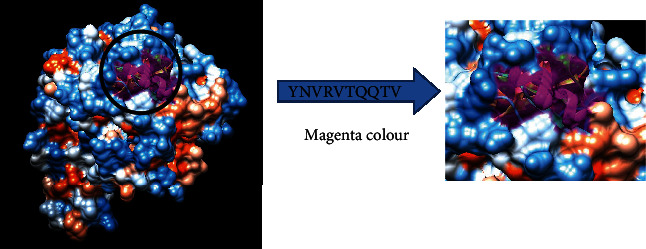
B cell epitopes proposed. The arrow shows the position of YNVRVTQQTV with Magenta colour in a structural level of fructose 1,6-bisphosphate aldolase. ^∗^The 3D structure was obtained using USCF Chimera software.

**Figure 7:**
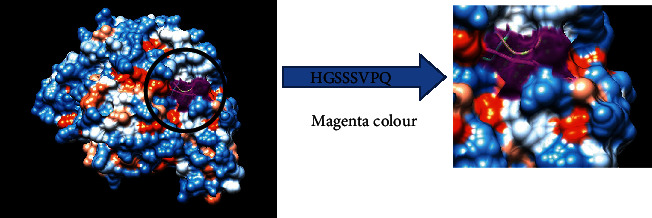
B cell epitopes proposed. The arrow shows the position of HGSSSVPQ with Magenta colour in a structural level of fructose 1,6-bisphosphate aldolase. ^∗^The 3D structure was obtained using USCF Chimera software.

The reference sequence of fructose 1,6-bisphosphate aldolase was subjected to BepiPred linear epitope prediction, Emini surface accessibility, Kolaskar and Tongaonkar antigenicity, and Parker hydrophilicity methods in the IEDB to test for various immunogenicity parameters (Table 2 and Figures 2–5). The tertiary structure of the proposed B cell epitopes is shown (Figures 6 and 7).

### 3.3. Prediction of Cytotoxic T Lymphocyte Epitopes and Interaction with MHC Class I

The reference fructose 1,6-bisphosphate aldolase sequence was analyzed using the (IEDB) MHC I binding prediction tool to predict T cell epitopes which suggested interacting with different types of MHC class I alleles, based on Artificial Neural Network (ANN) with half-maximal inhibitory concentration (IC50) < 500 nm. 206 peptides were predicted to interact with different MHC I alleles.

**Figure 8:**
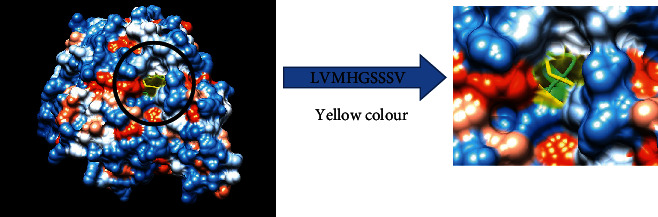
T cell epitopes proposed that interact with MHC I. The arrow shows the position of LVMHGSSSV with yellow colour in a structural level of fructose 1,6-bisphopsphate aldolase. ^∗^The 3D structure was obtained using USCF Chimera software.

The most promising epitopes and their corresponding MHC I alleles are shown in Table 3 along with the 3D structure of the proposed one (Figure 8).

### 3.4. Prediction of the T Cell Epitopes and Interaction with MHC Class II

**Figure 9:**
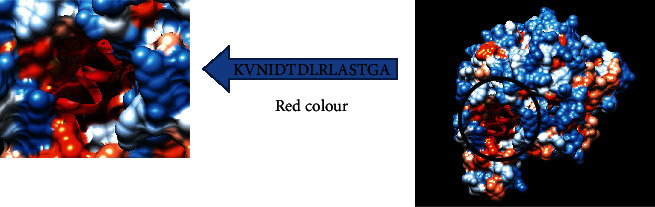
T cell epitopes proposed that interact with MHC II. The arrow shows the position of KVNIDTDLRLASTGA with red colour in a structural level of fructose 1,6-bisphosphate aldolase. ^∗^The 3D structure was obtained using USCF Chimera software.

The reference fructose 1,6-bisphosphate aldolase sequence was analyzed using the (IEDB) MHC II binding prediction tool based on NN-align with half-maximal inhibitory concentration (IC50) < 100 nm; there were 662 predicted epitopes found to interact with MHC II alleles. The most promising epitopes and their corresponding alleles are shown in (Table 4) along with the 3D structure of the proposed one (Figure 9)

### 3.5. Population Coverage Analysis

All promising MHC I and MHC II epitopes of fructose 1,6-bisphosphate aldolase were assessed for population coverage against the whole world (Table 5).

**Figure 10:**
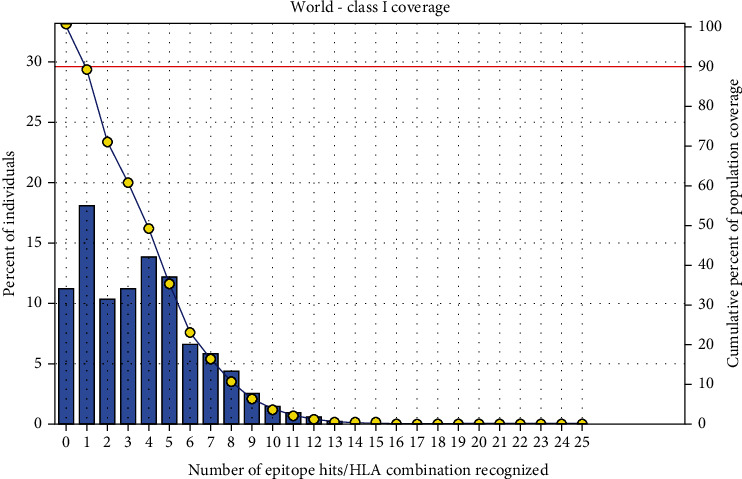
Population coverage for MHC class I epitopes.

**Figure 11:**
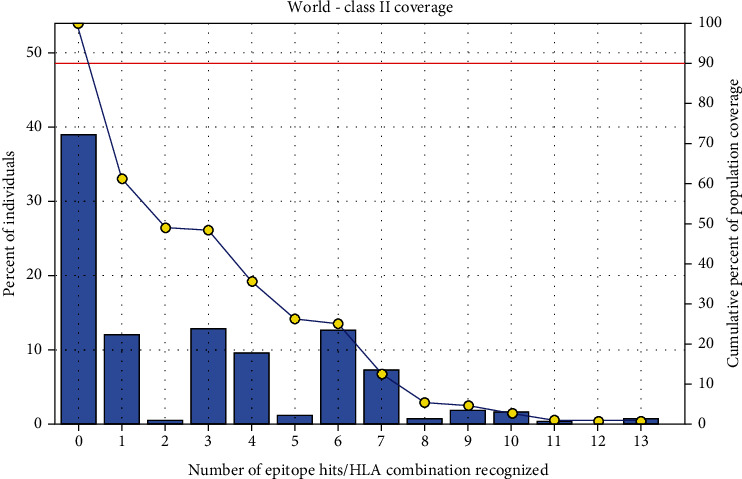
Population coverage for MHC class II epitopes.

**Figure 12:**
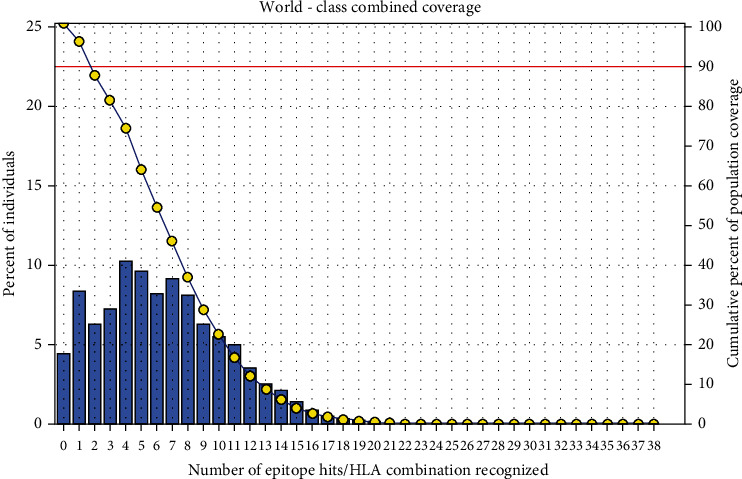
Population coverage for MHC class I and II epitopes combined.

For MHC I, epitopes with the highest population coverage were LVMHGSSSV (60.41%) and QMLDHAAEF (31.7%) (Figure 10 and Table 6). For MHC class II, the epitopes that showed the highest population coverage were KVNIDTDLRLASTGA (27.37%) and GEIKETYGVPVEEIV, GGEIKETYGVPVEEI, and YGGEIKETYGVPVEE (24.27%) (Figure 11 and Table 7). When combined together, the epitopes that showed the highest population coverage were LVMHGSSSV (60.41%), QMLDHAAEF (31.7%), and KVNIDTDLRLASTGA (27.37%) (Figure 12).

## 4. Discussion

Vaccination against *P. aeruginosa* is highly accredited due to the high mortality rates associated with the pathogen that spreads through healthcare areas. In addition, multidrug resistance of the pathogen demands the design of vaccine as an alternative [43]. In this study, immunoinformatics approaches were used to propose different peptides against FBA of P. aeruginosa for the first time. These peptides can be recognized by B cell and T cell to produce antibodies. Peptide vaccines overcome the side effects of conventional vaccines through easy production, effective stimulation of immune response, less allergy, and no potential infection possibilities [35]. Thus, the combination of humoural and cellular immunity is more promising at clearing bacterial infections than humoural or cellular immunity alone.

As B cells play a critical role in adaptive immunity, the reference sequence of *P. Aeruginosa* FBA was subjected to BepiPred linear epitope prediction 2 test to determine the binding to B cell, Emini surface accessibility test to test the surface accessibility, Kolaskar and Tongaonkar antigenicity test for antigenicity, and Parker hydrophilicity test for the hydrophilicity of the B cell epitope.

Out of the thirteen predicted epitopes using BepiPred 2 test, only three epitopes passed the other three tests (ADKTDSPVI, YNVRVTQQTV, and HGSSSVPQ) after segmentation. BepiPred version 2 test was used because it implements random forest and therefore predicts large epitope segments.

The reference sequence was analyzed using the IEDB MHC I and II binding prediction tools to predict T cell epitopes. 28 epitopes were predicted to interact with MHC I alleles with half-maximal inhibitory concentration (IC50) < 500. Six of them were most promising and had the affinity to bind to the highest number of MHC I alleles (LVMHGSSSV, QMLDHAAEF, AIGTSHGAY, GTSHGAYKF, IQLGFSSVM, and ISLEGMFQR). 19 predicted epitopes were interacted with MHC II alleles with IC50 < 100. Four of them were most promising and had the affinity to bind to the highest number of MHC II alleles (GEIKETYGVPVEEIV, GGEIKETYGVPVEEI, KVNIDTDLRLASTGA, and YGGEIKETYGVPVEE). Nineteen epitopes (NVNNLEQMR, IQLGFSSVM, AADKTDSPV, SIQLGFSSV, GEIKETYGV, AIGTSHGAY, VPAFNVNNL, KVNIDTDLR, LAIAIGTSH, IVQASAGAR, ETYGVPVEE, GTSHGAYKF, YGGEIKETY, VIVQASAGA, IAIGTSHGA, RKVNIDTDL, FNVNNLEQM, YGVPVEEIV, and SPVIVQASA) appeared in both MHC I and II results.

The best epitope with the highest population coverage for MHC I was LVMHGSSSV (60.41%) with seven HLA hits, and the coverage of population set for the whole MHC I epitopes was 88.75%. Excluding certain alleles for MHC II, the best epitope was KVNIDTDLRLASTGA scoring 27.37% with two HLA hits, followed by GEIKETYGVPVEEIV scoring 24.27% with five HLA hits. The population coverage was 61.1% for all conserved MHC II epitopes. These epitopes have the ability to induce T cell immune response when interacting strongly with MHC I and MHC II alleles effectively generating cellular and humoural immune response against the invading pathogen. When combined, the epitope LVMHGSSSV had the highest population coverage percent 60.41% with seven HLA hits for both MHC I and MHC II.

Many studies had predicted peptide vaccines for different microorganisms such as rubella, Ebola, dengue, Zika, HPV, Lagos rabies virus, and mycetoma using immunoinformatics tools [44–51]. Limitations include the exclusion of certain HLA alleles for MHC II.

We hope that the world will benefit from these predicted epitopes in the formulation of the peptide-based vaccine and recommend further *in vivo* and *in vitro* studies to prove its effectiveness along with formulation of appropriate adjuvants. Finding another immunogenic target and analyzing the associated epitopes support the vaccine formula.

## 5. Conclusion

Vaccination is used to protect and minimize the possibility of infection leading to an increased life expectancy. The design of vaccines using immunoinformatics prediction methods is highly appreciated due to the significant reduction in cost, time, effort, and resources. Epitope-based vaccines are expected to be more immunogenic and less allergenic than traditional biochemical vaccines. We have illustrated different epitopes that have the ability to stimulate both B and T cells against fructose bisphosphate aldolase protein of *Pseudomonas aeruginosa* for the first time. Three B cell epitopes have successfully passed the required tests. Six MHC I epitopes were found to be most promising, while four were found from MHC II epitope result set. These epitopes covered 95.62% worldwide excluding certain MHC II alleles.

## Figures and Tables

**Table 1:** Molecular weight and amino acid frequency distribution of the protein.

Amino acid	Number	Mol%
Ala	37	10.45
Cys	4	1.13
Asp	22	6.21
Glu	26	7.34
Phe	13	3.67
Gly	31	8.76
His	11	3.11
Ile	24	6.78
Lys	17	4.8
Leu	23	6.5
Met	12	3.39
Asn	9	2.54
Pro	17	4.8
Gln	13	3.67
Arg	18	5.08
Ser	21	5.93
Thr	18	5.08
Val	26	7.34
Trp	1	0.28
Tyr	11	3.11

**Table 2:** List of conserved peptides with their antigenicity, Emini surface accessibility, and Parker hydrophilicity scores (^∗^peptides that successfully passed the three tests).

Peptide	Start	End	Length	Kolaskar & Tongaonkar antigenicity score (TH: 1.025)	Emini surface accessibility score (TH: 1)	Parker hydrophilicity prediction score (TH: 1.681)
RQMLDHAA	7	14	8	1.008	1.013	1.637
FNVNNLEQMRAIM	23	35	13	0.974	0.432	0.2
AADKTDSPVIVQASAGARK	37	55	19	1.031	0.776	3.3
ADKTDSPVI^∗^	38	46	9	1.027	1.084	3.322
MHQDHGTSPDVCQ	80	92	13	1.035	0.868	3.785
SIQLGFSSVMMDGSL	94	108	15	1.03	0.082	0.38
EDGKTP	110	115	6	0.916	3.211	6.083
YNVRVTQQTVA	120	130	11	1.079	0.972	2.064
YNVRVTQQTV^∗^	120	129	10	1.081	1.229	2.06
AHACGVSVEGELGCLGSLETGM	132	153	22	1.057	0.011	1.818
GEEDG	155	159	5	0.863	1.532	7.4
GAEGVLDHSQ	161	170	10	1.029	0.539	3.3
LTDPEE	172	177	6	0.965	2.317	3.95
DALAIAIGTSHGAY	188	201	14	1.044	0.109	1.179
THLVMHGSSSVPQ	227	239	13	1.073	0.369	1.685
HGSSSVPQ^∗^	232	239	8	1.06	1.016	3.963
WLAII	241	245	5	1.102	0.134	-6.62
YGGEIKETYG	248	257	10	0.964	1.408	3.3
KVNIDTDLRLAST	273	285	13	1.018	0.843	1.985
AMRD	311	314	4	0.907	1.279	3.025
GTAGN	324	328	5	0.899	0.717	5.14
GEL	347	349	3	0.992	0.694	1.433

**Table 3:** The most promising T cell epitopes and their corresponding MHC I alleles.

Peptide	MHC I alleles
AADKTDSPV	HLA-C∗05:01, HLA-C∗03:03
AAIEEFPHI	HLA-A∗02:06
AIGTSHGAY	HLA-A∗30:02, HLA-B∗15:01, HLA-A∗29:02
ETYGVPVEE	HLA-A∗68:02
FNVNNLEQM	HLA-C∗12:03
GEIKETYGV	HLA-B∗40:02, HLA-B∗40:01
GELGCLGSL	HLA-B∗40:01, HLA-B∗40:02
GTSHGAYKF	HLA-A∗29:02, HLA-A∗32:01, HLA-B∗58
IAIGTSHGA	HLA-A∗02:06
IEEFPHIPV	HLA-B∗40:01
IQLGFSSVM	HLA-B∗15:01, HLA-A∗02:06, HLA-B∗15:02
ISLEGMFQR	HLA-A∗31:01, HLA-A∗68:01, HLA-A∗11:01
IVQASAGAR	HLA-A∗31:01, HLA-A∗68:01
KPISLEGMF	HLA-B∗35:01, HLA-B∗07:02
KVNIDTDLR	HLA-A∗31:01
LAIAIGTSH	HLA-B∗35:01, HLA-C∗03:03
LVMHGSSSV	HLA-A∗02:06, HLA-A∗68:02, HLA-C∗12:03, HLA-C∗14:02, HLA-A∗02:01
NVNNLEQMR	HLA-A∗68:01
NVRVTQQTV	HLA-A∗30:01
QMLDHAAEF	HLA-A∗02:06, HLA-A∗29:02, HLA-B∗15:01, HLA-B∗15:02, HLA-A∗32:01
RKVNIDTDL	HLA-B∗48:01
SIQLGFSSV	HLA-A∗02:06
SLEGMFQRY	HLA-A∗29:02, HLA-A∗30:02
SPVIVQASA	HLA-B∗07:02
VIVQASAGA	HLA-A∗02:06
VPAFNVNNL	HLA-B∗07:02
YGGEIKETY	HLA-C∗12:03
YGVPVEEIV	HLA-C∗12:03

**Table 4:** The most promising T cell epitopes and their corresponding MHC II alleles.

Peptide	MHC II alleles
KVNIDTDLRLASTGA	HLA-DRB1∗03:01, HLA-DRB1∗11:01
GEIKETYGVPVEEIV	HLA-DRB1∗07:01, HLA-DRB1∗13:02, HLA-DQA1∗05:01/DQB1∗02:01, HLA-DQA1∗04:01/DQB1∗04:02, HLA-DQA1∗03:01/DQB1∗03:02
GGEIKETYGVPVEEI	HLA-DRB1∗07:01, HLA-DRB1∗13:02, HLA-DQA1∗04:01/DQB1∗04:02
	HLA-DQA1∗03:01/DQB1∗03:02
YGGEIKETYGVPVEE	HLA-DRB1∗07:01, HLA-DRB1∗13:02

**Table 5:** The population coverage of the whole world for the most promising epitopes of MHC I, MHC II, and MHC I and II combined.

Country	MHC I	MHC II	MHC I,II (combined)
World	88.75%	61.1%^∗^	95.62%^∗^

^∗^In the population coverage analysis of MHC II; 8 alleles were not included in the calculation; therefore, the above (^∗^) percentages are for epitope sets excluding these alleles: HLA-DQA1∗05:01/DQB1∗03:01, HLA-DQA1∗01:02/DQB1∗06:02, HLA-DQA1∗03:01/DQB1∗03:02, HLA-DRB4∗01:01, HLA-DRB5∗01:01, HLA-DQA1∗05:01/DQB1∗02:01, HLA-DPA1∗03:01/DPB1∗04:02, HLA-DQA1∗04:01/DQB1∗04:02.

**Table 6:** Population coverage of the proposed peptide interaction with MHC class I.

Epitope	Coverage (%)	Total hits
LVMHGSSSV	60.41	7
QMLDHAAEF	31.70	8
ISLEGMFQR	25.64	3
KPISLEGMF	20.62	2
LAIAIGTSH	15.85	2

**Table 7:** Population coverage of proposed peptides interaction with MHC class II.

Epitope	Coverage (%)	Total hits
KVNIDTDLRLASTGA	27.37%	2
GEIKETYGVPVEEIV	24.27%	5
GGEIKETYGVPVEEI	24.27%	4
YGGEIKETYGVPVEE	24.27%	2
GVRKVNIDTDLRLAS	23.90%	2

## Data Availability

The data which support our findings in this study are available from the corresponding author upon reasonable request.
